# Years of Life Lost and Childhood and Adolescent Cancer Mortality in Yazd Province, Iran (2004-2009)

**Published:** 2015-07-20

**Authors:** M Mirzadeh, M Mirzaei, M Mirzaei, H ShogaeiFar

**Affiliations:** 1 Department of Internal medicine, School of Medicine- Shahid Sadoughi University of Medical Sciences and Health Services, Yazd, Iran; 2 Department of community medicine, School of Medicine-Shahid Sadoughi University of Medical Sciences and Health Services, Yazd, Iran.; 3 Tehran University of Medical Sciences and Health Services, Tehran, Iran.; 4 Health Center of Yazd province- Shahid Sadoughi University of Medical Sciences and Health Services, Yazd, Iran.

**Keywords:** Years of Life Lost, Cancer, Mortality, Childhood, Iran

## Abstract

**Background:**

The Years of Life Lost (YLL) due to cancer can be a more illustrative index to promote preventive and therapeutic services, this study aimed to investigate the childhood cancer mortality and its trend over the past few years in Yazd to provide planners with baseline data.

**Material and Method:**

Data obtained from death registration system of the healthcare center were used to calculate the age-specific standardized mortality for 5-year age groups. To calculate the YLL, the standardized expected YLL method was used with a discount rate of 0.03 for health gain in the future, the age weight of 0.04 for different years of age, and a constant age weight correction factor of 0.165. The collected data were analyzed using the Epi 6 and Excel 2007.

**Results:**

28 patients in Yazd aged 0-19 die annually due Leukemia and CNS tumors were the most common causes of death. The crude cancer death rate is 8.48 in boys and 6.72 in girls per 100000. Premature cancer deaths have caused 3,436 YLL in boys and 2,561 YLL in girls (4.92% of total YLL in both sex) .The average death age did not have a significant relationship with sex or location of residence.

**Conclusion:**

Childhood cancer mortality has had a decreasing trend. This study can help in the assessment of healthcare needs and improvement of the quality of healthcare services. It can also help in the design and prioritization of interventions to identify cancer risk factors which can facilitate early diagnosis.

## Introduction

Despite the success in the prevention and control of communicable diseases, especially in childhood, in recent decades, the prevalence of chronic diseases is increasing (1). According to WHO’s estimation, the prevalence of cancer in childhood is 100 in 1,000,000 (2). Although cancer is less prevalent in children compared to adults and constitutes less than one percent of all cancers (3-5), one American child is diagnosed with cancer in each 285 children before the age of 20 (6). In other words, an American child has 0.35% chance of being diagnosed with cancer before the age of 20 (7). At the ages of 0-19, cancer in the fourth and the second leading cause of death among American boys and girls (8-9), and despite progress in the treatment and reduction of mortality and increase in the five-year survival rate (10), more than 20% of children die, and a significant percentage of the survivors do not have a high quality life (11).

The total incidence of cancer among Iranian children varies by geographical regions so that it is reported 48-112 and 51-144 per 1,000,000 for girls and boys, respectively (12).

Cause-specific mortality rate is a basic calculation in a healthcare system. Regarding cancer, calculations of mortality trend can indicate the effects of screening plans, early diagnosis and treatment, and the environmental risk factors in cancer incidence and control. Since cause-specific mortality may not be a proper index for plan prioritization and resource allocation due to common causes of death in old age (13), measuring years of life lost, especially in childhood and adolescence can help policy-makers prioritize health problems and design healthcare interventions (14). According to census in 2006 and 2011, the population of Yazd province was 990,818 (36.7% under 20 years of age) and 1,074,428 (33.1% under 20 years of age) (15). Therefore, given that one-third of the population of Yazd province is under 20 years of age, this study aimed at calculating the years of life lost caused by cancer in children in Yazd and describing the existing condition in order to determine baseline information for policy-making decisions so that by periodically calculating this index, the outcomes of implementation of the designed interventions in disease treatment and risk factor control can be assessed.

## Materials and Methods

The studied population consisted of all individuals in Yazd province during 2004-2009. Urban population was obtained based on the base population, which was obtained from the census of the year 2006, using annual population growth and exponential method. Rural population was calculated using vital horoscope completed annually in healthcare centers. Childhood cancer mortality was obtained using the death registration system of the Yazd healthcare center. Death registration data was recorded according to the standard death certificate from all available diagnosis sources such as hospitals, cemeteries, forensic medicine centers, healthcare centers, and other resources throughout the city. Age-specific standardized mortality for 5-year age groups was calculated using the direct method. To make the study comparable to the literature, the methodology of the original global burden of disease research conducted in 2003 (16) was used here. To calculate the years of life lost, standardized expected years of life lost method was used by the discount rate of 0.03 for future, age weight of 0.04 for different years of age, and an age weight correction factor of 0.165 (17). The collected data were analyzed using the Epi 6 and Excel 2007. 

## Results

**Figure 1:**
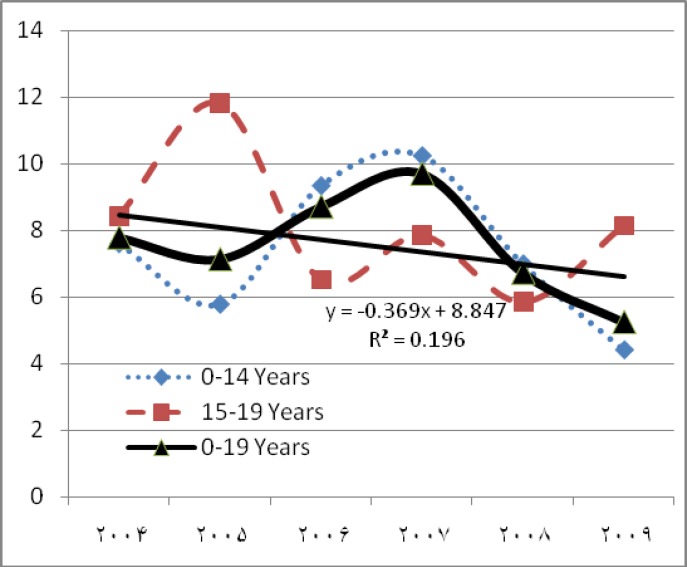
Trend of cancer mortality (standardized rate per 100,000 populations 0-19 years old) in Yazd Province, from 1383 to 1388

**Figure 2:**
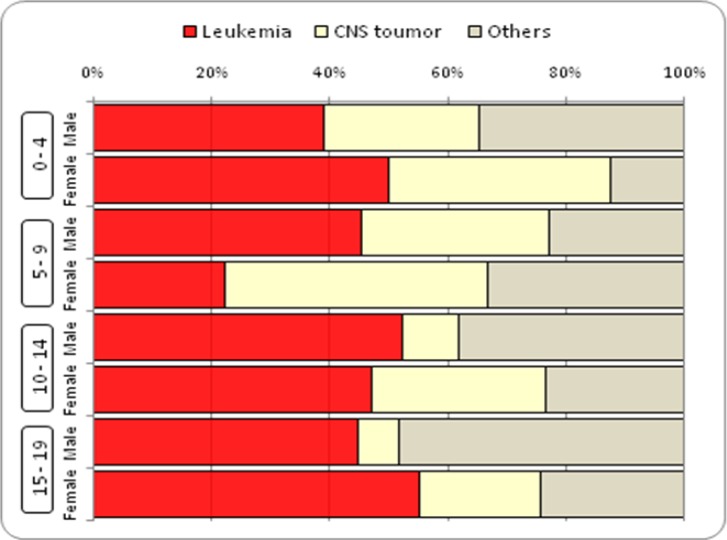
Percent distribution of childhood cancer mortality by type and age group, age <20 both sexes, Yazd. 1383-1388

During 2004-2009, 190 child deaths due to cancer were registered. Twenty-four of the children were excluded from the analysis since they resided in other provinces. Twenty-eight 0- to 19-year old individuals die of cancer annually in Yazd. In this study, 166 deaths were recorded during the research period (95 boys and 71 girls; male to female ratio 1.34) which indicated that 4.82% of all deaths in this age group was due to cancer. That percentage is slightly lower in boys compared to girls (4.85% in boys and 5.19% in girls). The crude cancer mortality was 8.48 per 100,000 in boys and 6.72 per 100,000 in girls. The average death age in boys and girls were 9.87 and 11.42, respectively. Among the most common cancers, the average age of the children diagnosed with central nervous system cancer, leukemia, bone cancer, and lymphoma at the time of death were 8.5, 10.9, 13.3, and 14, respectively (P value=0.006). The average death age did not have a statistically significant relationship with sex, location of residence, and the year of death. The age group of 15-19 with 34.9% had the highest mortality percentage, while the age group of 5-9 had the lowest mortality percentage (Table 1). In total, 3,436 years of life lost in boys, and 2,561 years of life lost in girls were due to cancer. The total years of life lost due to all causes for the 0-19 age group in the research period was estimated as 122,856, 4.92% of which was caused by cancer. The ratio was higher for girls compared to boys (5.3% compared to 4.7%). The childhood cancer mortality in Yazd had a decreasing trend in the research period. The decrease was more in the 0-14 age group (7.6 to 4.4 per 100,000) compared to the 15-19 age group (8.44 to 8.15 per 100,000) (Figure 1). Different types of leukemia and central nervous system tumors were the most common causes of childhood cancer mortality. Leukemia was the most common cause of death in all age groups except the girls in the 5-9 age group. Figure 2 shows the cause of cancer deaths by age-sex groups and by the common causes.

**Table I:** Childhood Cancer Mortality & Years of Life Lost by Sex, age at death and cancer type, Yazd, 1383-1388

	**YLL** ** (Years)**	**Mortality Crude Rate/100000**	**Death Number(%)**
Total	5997.8	7.63	166 (100)
Sex			
Boys	3436.4	8.48	95 ( 57.2 )
Girls	2561.4	6.72	71 ( 42.8 )
Age of Death			
0-4	1328.2	7.82	39 ( 23.5 )
5-9	1155	6.84	31 ( 18.7 )
10-14	1420.8	7.34	38 ( 22.9 )
15-19	2093.8	8.20	58 ( 34.9 )
Cancer type ICD-10: C00–97			
Leukaemia ( C91–C95 )	2784	3.54	77 ( 46.4 )
Lymphoma ( C81–85 )	293.7	0.37	8 ( 4.8 )
CNS tumour ( C70–C72 )	1368.8	1.75	38 ( 22.9 )
Bone tumour ( C40–C41 )	257.9	0.32	7 ( 4.2 )
Others	1293.2	1.65	36 ( 21.7 )
Year			
1383	1092	7.85	30
1384	1049.3	7.78	29
1385	1125.1	8.52	31
1386	1178.3	9.24	33
1387	830.9	6.67	23
1388	722.2	5.61	20

YLL = Years of Life Lost, CNS = central nervous system. Other includes cancer sites with one or two deaths, as well as cancers of unspecified parts.

## Discussion

Comparison of cancer mortality in Yazd and Iran shows that in the research period, the childhood cancer mortality rate was higher in Yazd compared to Iran (7.37 compared to 4.6 per 100,000) (12). International comparison of Yazdi-Iranian childhood cancer mortality cancer shows that the rate is closer to Southern-Eastern Europe and is higher than Western-Northern Europe and the U.S. (18-19).

In this study, the highest cancer mortality belonged to leukemia and cancers of the central nervous system which is consistent with the results of other studies in developed countries (19-21) and inconsistent with the results obtained in Africa where lymphoma has the highest mortality among cancers (22-23). This fact indicates the impact of environmental risk factors and genetic differences in cancer incidence and mortality in the world. Given the geographical differences in Iran and in the world and also known effect of some environmental factors, such as father’s occupation, in the incidence of cancer, especially leukemia and lymphoma (24), further studies are required. Studies can help identify risk factors to identify patients at early stages and factors influencing survival of patients in Yazd despite reports of the suitability of the response to treatment in medical centers (25).

The declining trend of childhood cancer mortality in the research period is consistent with studies in several countries. The trend shows steeper decline in 0-14 age group (y=-0.326x + 8.535, r^2^ = 0.078) compared to 15-19 age group ( y=-0.514x + 9.911, r^2^ = 0.215 ) (19,21,26,27). Due to the differences in the results of this study with American children (11), it is necessary that similar studies with regard to age at diagnosis and duration disease be conducted to assess the effects of therapeutic interventions in 5-year survival rate and review continuity of care at a later age (28) and determine the probability of a shift in time of death to an older age in Yazd. The decreasing trend of cancer deaths was less significant in boys compared to girls. Given the social and cultural differences between the sexes in families in Yazd, it is suggested that these studies be continued in the coming years and the researchers address the differences in exposure to environmental risk factors for boys and girls in Yazd. The results can be used to design preventive interventions, especially since studies have shown that 5-year survival and prognosis of leukemia, which is a common cancer, is not significantly associated with sex after diagnosis (25, 29). Like anywhere in the world (19,23,26,30), the most significant reason of declining trend in childhood cancer mortality in Yazd is the improvements in the diagnostic-therapeutic facilities in the Yazd province and access to new protocols for leukemia and lymphatic system cancer during recent years. However, the decline in trend of central nervous system cancers mortality is not satisfactory since early detection and treatment methods and 5-year survival has remained unimproved. In contrast to a less significant declining trend in the central nervous system cancer ( y = -0.078x + 2.020 , r^2^ = 0.12 ), declining trend in leukemia ( y=-0.205x + 4.244 , r^2^ = 0.19 ) is inconsistent with reported decline in countries such as America, Italy, and England and is consistent with the findings from Australia and Japan (11,21,31). The analytical study of the incidence of cancer in children over the years will help explain these differences.

Given the existence of the death registration system and referral hospitals in Yazd in the treatment of disease, we can be sure that the results are able to show the cancer death characteristics in children in Yazd. However, due to the low number of deaths during our study, cause-specific estimates of cancer mortality have a wider confidence range. Therefore, continuing study about cancer mortality rates over the next few years along, the incidence of new cases, 5-year survival, and the comparisons of the trends with other causes of death in this age group can better illustrate the change (decrease/increase) in the mortality which is affected by prevention and treatment of cancer.

## Conclusion

This study presents the latest results of childhood cancer mortality in Yazd and can help in assessing patients' needs and developing intervention programs to improve the quality of treatment services for children with cancer. Moreover, it can direct health system administrators to design and prioritize action plans to identify risk factors and make early diagnosis to reverse and reduce childhood cancer disability and mortality.
